# Recent Advances in Metallic Nanoparticle Assemblies for Surface-Enhanced Spectroscopy

**DOI:** 10.3390/ijms23010291

**Published:** 2021-12-28

**Authors:** Beata Tim, Paulina Błaszkiewicz, Michał Kotkowiak

**Affiliations:** Faculty of Materials Engineering and Technical Physics, Poznan University of Technology, Piotrowo 3, 60-965 Poznan, Poland; beata.tim@doctorate.put.poznan.pl (B.T.); paulina.blaszkiewicz@put.poznan.pl (P.B.)

**Keywords:** plasmonic, functionalization, surface-enhanced spectroscopy

## Abstract

Robust and versatile strategies for the development of functional nanostructured materials often focus on assemblies of metallic nanoparticles. Research interest in such assemblies arises due to their potential applications in the fields of photonics and sensing. Metallic nanoparticles have received considerable recent attention due to their connection to the widely studied phenomenon of localized surface plasmon resonance. For instance, plasmonic hot spots can be observed within their assemblies. A useful form of spectroscopy is based on surface-enhanced Raman scattering (SERS). This phenomenon is a commonly used in sensing techniques, and it works using the principle that scattered inelastic light can be greatly enhanced at a surface. However, further research is required to enable improvements to the SERS techniques. For example, one question that remains open is how to design uniform, highly reproducible, and efficiently enhancing substrates of metallic nanoparticles with high structural precision. In this review, a general overview on nanoparticle functionalization and the impact on nanoparticle assembly is provided, alongside an examination of their applications in surface-enhanced Raman spectroscopy.

## 1. Introduction

**Figure 1:**
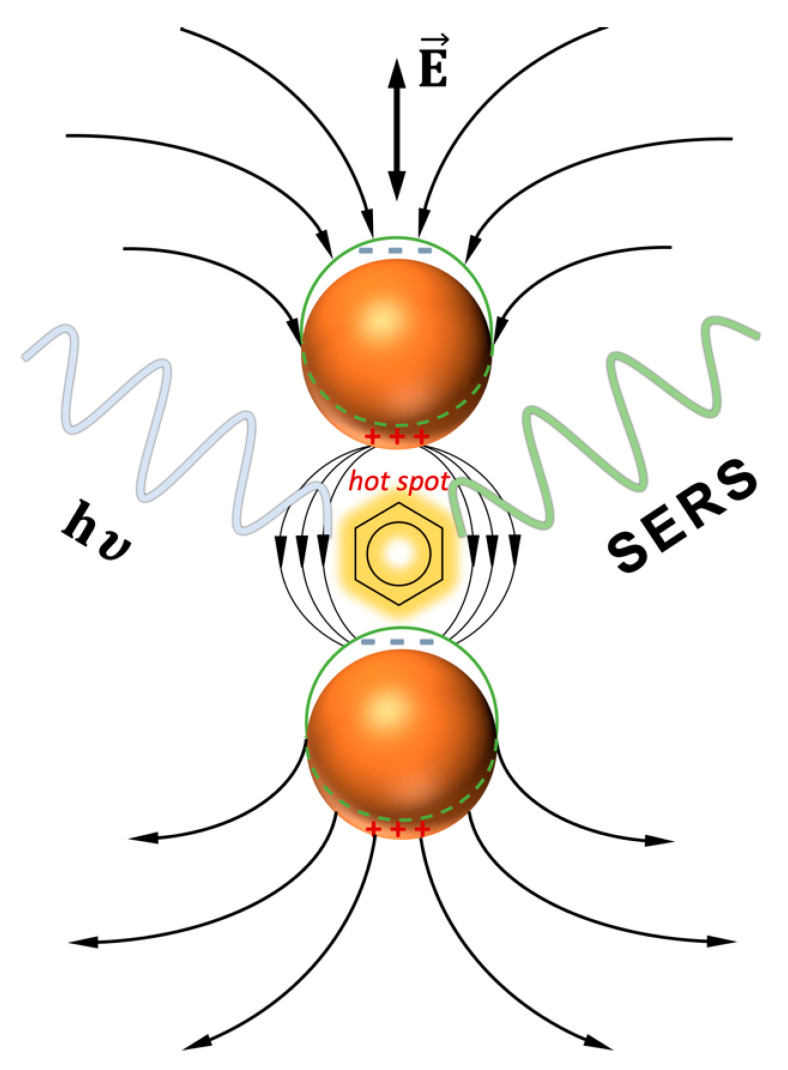
Scheme of the surface-enhanced Raman scattering (SERS) resulting from the electromagnetic effect. Image not to scale.

Delocalized electrons in noble metal nanoparticles (NPs) can easily oscillate when exposed to incident light. Such surface-charge-density oscillations may cause electric field enhancements close to the surface of NPs. Particles of this type deserve special attention due to their ability to form surface plasmon resonances, which are responsible for their optical properties [1,2,3,4]. Resonant excitation of free electrons by input of electromagnetic radiation at a particular wavelength generates surface plasmons, which are related to strong characteristic bands in the extinction spectrum (namely, the LSPR band). Depending on their shape and size, NPs can exhibit plasmonic resonance in different regions of their spectra. One-dimensional assemblies of NPs are a particularly interesting area of research due to their potential applications in electronics [5], photonics [6], and sensing [7]. Most of the current work is performed with the use of self-assembly properties and the effects of the metallic surfaces. The process of self-organization is the spontaneous formation of NPs into stable configurations without covalent bond interactions [8,9]. Near-field enhancement is created by Raman and fluorescence spectroscopy techniques, by improving their detection sensitivities. The two phenomena, in which the unique plasmonic properties of the noble metal NPs can play a substantial role, are surface-enhanced Raman scattering (SERS) and metal-enhanced fluorescence (MEF) [10]. During the past few years, the ability to use bottom-up NP assemblies to produce small-sized gaps in a reproducible and a robust manner has opened further opportunities for the application of SERS. When a molecule is within close proximity of a surface of NPs, it can absorb or sometimes radiate a greater amount of light. This happens due to the fact that both the absorption and emission can occur with certain probabilities, which can be modified by nearby NPs [11]. This phenomenon is widely used in the development of functional nanoplatforms for biosensing applications, due to the ability of plasmon resonance to enhance the scattered signals from the adsorbed biomolecules [12,13]. Raman spectroscopy, including its surface-enhanced form, is one of the most frequently used methods for identify a molecule [14,15]. Plasmonic effects can increase, by a few orders of magnitude, the signal of an otherwise very inefficient process. The key aspect relating to the efficiency of SERS from the substrates is the presence of plasmonic hot spots at specific sites in which the input light is trapped [14,16]. A scheme of the surface-enhanced Raman scattering (SERS) resulting from the electromagnetic effect is shown in Figure 1. The easiest approach for the preparation of SERS active platforms is based on the formation of self-assembly monolayers (SAMs) composed of NPs [17,18]. The main limitation of this technique, largely due to steric hindrance, is that the surface coverage can only reach approximately 50%. Unfortunately, this low surface coverage reduces the hot spots density and, thus, the SERS response [15,16,19,20]. However, the latter is controllable by increasing and tuning the density of the hot spots on the substrate. The SERS active substrates can be obtained in two different ways. The NPs either aggregate in a solution or are deposited at the solid substrates. The latter is generally more stable and easier to handle; however, in most cases, this mode of production is expensive, and advanced physicochemical methods should be used, instead, in their creation. As well as simple strategies for NP assemblies, more advanced protocols have recently been introduced.

An efficient and scalable bottom-up strategy for the development of nanostructured materials is based on the self-assembly of NPs via wet chemistry methods [21,22]. A particularly versatile approach is the use of NPs with sharp edges, for example, nanoprisms (NPRs) or nanostars (NSs) [23,24,25]. The synthesis methods for monodisperse NPs are well established [26]. However, despite the proposed approaches in the literature resulting in stable NPs, large sized NPs and the types of ligands that stabilize such large structures can lead to difficulties in the functionalization of the produced NPs for specific applications [18]. Therefore, although there is a significant amount of literature on the synthesis of NPs already, there remains a strong need for the development of a robust protocol for creating more efficient methods for NP functionalization. In other words, typically, the prepared NPs require further modifications to closely control their properties [27]. The Langmuir technique is an excellent method for depositing self-assembled systems onto surfaces. It offers homogeneity over relatively large areas, and unlike traditional SAMs, multiple layers of films can be created by successive dipping [28]. However, Langmuir monolayers can only be produced from materials that are soluble in non-polar solvents and immiscible with the polar subphase (water), meaning that reliable methods for NP transfer are required to ensure transfer from water to organic solvents such as chloroform. A rapid procedure has recently been proposed, based on a combination of the commercially available thiolated poly(ethylene glycol) (PEG-SH) and 1-dodecanethiol, which acts as a general method for transferring a wide variety of gold and silver NPs with different sizes and shapes, such as spherical NPs, nanorods (NRs), and NSs, from aqueous dispersion into chloroform [29].

During the last few decades, the field of SERS has seen a great upturn in progress. However, a very limited number of reviews relating to metallic NP assemblies are found in the literature. Thus, to help rectify this shortage, this review provides an overview of the recent developments in NP functionalization and assesses the impact of NP assembly on SERS. To begin, we describe methods for the functionalization of the surface of the NPs and how this functionalization affects the properties of the NPs. Next, we analyze assemblies of anisotropic NPs and examine their applications, with particular emphasis on NRs, NSs, NPRs, and nanocubes (NCs). The final section is a presentation on the recent developments of spherical NP assemblies which, due to the simple method for their synthesis, are widely used in SERS applications.

## 2. Surface Functionalization of Metallic Nanoparticles

There are many different methods for synthesizing NPs with different sizes and shapes. However, the control of these shapes and sizes, which is an important aspect of the synthesis, can be difficult [30]. This is especially unfortunate because the surface activity and the optical and electrical properties of the NPs are dependent on them [27,31]. Moreover, in the case of Au-NR synthesis, the lack of control over the size of the nanorods results in the formation of a large number of Au-NPs, which impairs the entire synthesis process [32,33]. One way to control the shapes of the NPs is via compounds that block the growth of certain crystallographic walls. The most commonly used compounds for this purpose are sodium dodecyl sulfate and cetyltrimethylamine bromide (CTAB) [34,35]. However, it is known that CTAB is a toxic compound, which means that its use is unfavorable in connection to the production of NPs for biomedical purposes. Since CTAB can reduce the viability of cells by up to 50%, efforts have been made to develop alternative NP synthesis methods that do not rely on cytotoxic compounds or replace the stabilizing ligand with one that does not have toxic properties for cell lines [36,37].

It has been proven that NPs have a relatively large aspect (length-to-width) ratio when obtained using aqueous seed solutions. The charge on the surrounding surfactant stabilizes the surface of the NPs, which is located at the nucleus, and affects the aspect ratio; a positive charge produces a more noticeable effect than a negative one [38,39]. After the synthesis of the NPs via physical and chemical processes, the functionalization process is the next preparation stage. This can also be performed in situ, simultaneously to the production of the NPs. Functionalization improves the stability of the NPs in organic solvents, and it prevents agglomeration. However, functionalization is not always performed to prevent agglomeration, sometimes controlled agglomeration is needed to build appropriate systems, for example, based on catalytic, adsorption, and electronic effects [40]. Functionalization increases the cost of the obtained nanostructures by up to 10–50%, but it is usually necessary. This is because NPs can undergo an oxidation process, therefore functionalization is required to protect the material from surrounding environmental influences.

An important stage after the functionalization process is the maintenance of structural stability, for which the functional agent needs to induce sufficient repulsions between the NPs. Another significant issue, after the functionalization process, is to preserve the morphology and shape of NPs. The purpose of functionalization is to obtain stable and highly monodispersed NPs, which results from an optimization process. There are many techniques for the functionalization of NPs, for example, via the use of receptors [41] and similar biomolecules, which are, thus, ideal candidates for biological applications. Functionalization and stabilization of nanostructures can also be performed with the use of the following chemical compounds: CTAB surfactant [42,43], silica dioxide [44], PEG-SH [45], titanium dioxide [46], bovine serum albumin [47], folic acid [48], polypeptides [49], oligonucleotides [50], antibodies [51], sodium citrate [52], and compounds with thiol [53]. In addition, citrate-stabilized NPs are more accessible, and it is easier for further functionalization compared to NPs functionalized with (polyvinylpyrrolidone) PVP and CTAB. Therefore, bimetallic Au-Ag NPs that are embedded with citrates are used in the fabrication of nanoplatforms. Due to the possibility of easy coverage over a given surface with the functionalized NPs, it can become straightforward for the layers to contain the desired optical, electrical, or chemical properties.

Functionalization compounds are extremely attractive in many applications; the choice of the functional group used determines the surface properties, and the monolayer properties can be modified by the selection of the necessary chain length. The type of functional groups on the compounds that functionalize the self-organized monolayers can also modify the surface characteristics of a biosensor, which can bind to molecules by means of chemical or physical interactions. However, in such a scenario, chemical functionalization ensures greater system stability compared to physical interactions. The implementation of the chemical method leads to the possibility of the covalent bonding of the NPs to the ligands, either due to a direct reaction or due to indirect reactions via use of the appropriate linkers [54]. The functional groups of the organic or biological compounds determine both the method and nature of NP functionalization; however, there are other important factors to consider. For example, particular attention should be paid to the quality of the resulting monolayer [55]. The possible formation of monolayers with areas of heterogeneity or discontinuity suggests the superiority of multilayer coatings in such a case. However, the best results are obtained by use of good quality monolayers, which have the least variation in their properties across the surface. In contrast, multilayers formed by the physical adsorption of successive monolayers may have numerous aggregates, which would disturb the uniformity of the surface coverage. For all these reasons, functionalization is a very important process that allows the creation of stable NPs that maintain their morphology and chemical structure. Due to the increased interest in self-organized materials, the development of new functionalization schemes can be foreseen in the near future.

## 3. Assemblies of Noble Anisotropic Nanoparticles

### 3.1. Rod-Shaped Nanoparticles

Anisotropic NPs are characterized by the possibility of large electromagnetic field enhancements at the single-particle level. Consequently, anisotropic NPs have recently attained a lot of attention as a substrate for SERS studies. Au-NRs have more plasmonic hot spots than spherical NPs and, therefore, exhibit greater SERS enhancements [56]. According to previous research [15,56,57,58,59], substrates that consist of aggregated silver and gold colloids are characterized by improved SERS activity in comparison to systems composed of only one type of metal. However, the activity of bimetallic NPs is dependent on the molar ratio of Ag to Au, since a higher Au mole fraction means a greater intensity peak on the SERS spectra [60]. Guven et al. [61] presented a SERS method for the quantitative determination of nitrites (NO_2_^−^) from the alkaline hydrolysis of explosives. For this purpose, 4-aminothiophenol (4-ATP) modified Au-NRs, doped with Ag-NPs, were used as the substrate that increased the signal intensity. This paper was particularly noteworthy because the authors were the first to present a SERS study that quantified octahydro-1,3,5,7-tetranitro-1,3,5,7-tetrazocine and hexahydro-1,3,5-trinitro-1,3,5-triazine in synthetic solutions and real samples. In another study, Gomez-Grana et al. [62] used core-shell structures (Au@Ag-NRs) as supercrystalline SERS substrates with high electric field enhancements. For the fabrication of the tested material, a method to control the conditions of the drop casting was used; gemini surfactants were employed as the stabilizer. While benzenethiol (BT) was used as the SERS probe that confirmed the enhanced properties of the material. The authors determined that highly organized structures provide a greater sized SERS intensity compared to randomly distributed Au-NRs. Moreover, they also showed the influence of the geometrical orientation of the structures in the supercrystal on the reinforced signal. Standing crystals are characterized by a greater intensity compared to lying structures, which is due to the transfer of an electric field onto the supercrystal. Huang et al. [63] developed an efficient SERS substrate that consists of a bimetallic Au-Pd nanostructure. The material they established is characterized by a combination of both the plasmonic and catalytic activity within one crystal. The authors presented a SERS platform for the monitoring of the catalytic reactions in situ. In this innovative approach, to obtain the substrate, a procedure that consists of the selective growth of Au-Pd alloy horns onto the ends of single-crystal Au-NRs (denoted as HIF-Au-NR@AuPd) was used. The properties of the produced materials were checked via a SERS analysis for the reaction involving the Pd-catalyzed hydrogenation of 4-nitrothiophenol (4-NTP) to create 4-ATP. Initially, the 4-NTP molecules formed a monolayer on the HIF-Au-NR@AuPd surface. On this surface, after the introduction of the hydrogen flow, there was a reduction of the 4-NTP molecules to generate 4-ATP via the Pd catalyst. This research enabled a determination of the kinetics of the tested reaction: it proved that the hydrogenation of the monolayer of 4-NTP molecules on the HIF-Au-NR@AuPd surface proceeds according to first-order kinetics.

**Figure 2:**
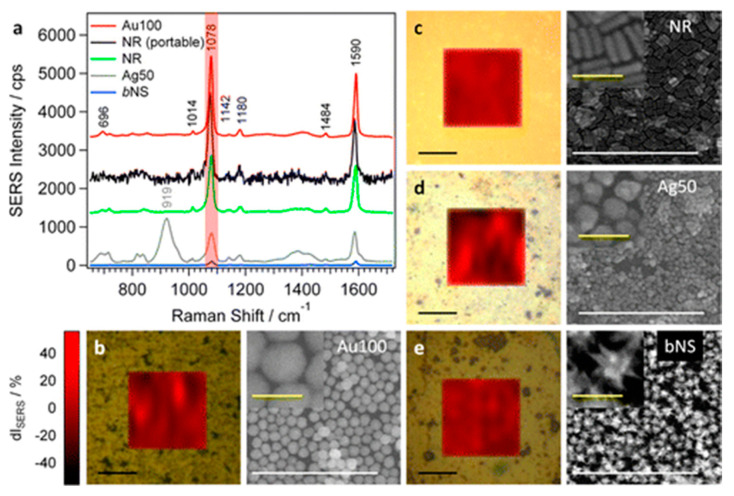
(**a**) Averaged SERS spectra of 4-mercaptobenzoic acid (10 μM) spherical nanoparticles (100 ± 10 nm) (**b**), gold nanorods (61 ± 5 nm length; 15 ± 2 nm width) (**c**), silver nanoparticles (51 ± 8 nm) (**d**), and big gold nanostars (56 ± 4 nm/98 ± 7 nm) (**e**). The corresponding SERS maps for the signal at 1078 cm^−1^ (red-shaded area in (**a**) as a function of the deviation from the average intensity dI (in %), together with representative SEM images of the assemblies before analyte incubation. The black SERS spectrum in (**a**) corresponds to the average of three 4-MBA spectra on the Au-NR sample measured with a portable Raman spectrometer. Reproduced with permission from [29], Langmuir; published by the American Chemical Society, 2015. Further permissions related to this material excerpted should be directed to the American Chemical Society.

SERS is also used in the diagnosis of diseases, including in cancer imaging. Alvarez-Puebla et al. [64] used highly organized supercrystals of Au-NRs, with plasmonic antennae, to detect prions in complex biological media such as serum and blood. Initially, the measurements were made using BT, on the basis of which good SERS properties of the NR supercrystals were found. The potential of the SERS medium to detect scrambled prions within complex biological media was then tested. For this purpose, the model peptide (106–126) (which is often used to investigate prion diseases), bovine serum, and human blood were used as the model substances [65]. The analysis showed that the three-dimensional organization of NRs into the supercrystals causes uniform electric field enhancements and high intensity hot spots, which means they have great potential as a SERS substrate. Torul et al. [66] used Au-NRs to detect blood glucose. This was achieved by the use of mixed SAM modified Au-NRs immobilized on mixed SAM modified gold surfaces, which were used as the substrate. The formation procedure of the latter was based on the development of a mixed monolayer of 3-mercaptophenylboronic acid and decanethiol groups on the surface of the gold-coated slide and on the surface of the Au-NPs. As a result of the interactions between the mixed monolayer and the glucose, a complex was formed that produced a decrease in the peak intensity that accompanied an increase in the glucose concentration. This enabled confirmation of the applicability of the tested platform as a sensor for glucose detection. Another interesting approach for the fabrication of SERS substrates was showed by Tsvetkov et al. [44]. The authors used nanopowders of Au-NRs to obtain concentrated Au-NRs sols, which were then deposited on an opal-like photonic crystal film formed on a silicon wafer. The substrates prepared in this manner are characterized by a multilayer structure and various spatial Au-NRs configurations. Rhodamine 6G (R6G) dye was applied to the surface of substrates, which contained various thicknesses. From the analysis, it was determined that the thickness of the substrate influences the intensity of the SERS spectra: a greater enhancement was observed for thinner substrates. The occurrence of this effect may result from the increased specific surface area and, thus, from the greater number of molecules that appear in the Raman spectra. In addition, the inhomogeneity of the Au-NR pattern on the substrates may provide more plasmonic hot spots. A study using R6G as the model molecule for SERS was also presented by Tim et al. [67]. This method involved the formation of nanoplatforms that were composed of pegylated Au-NRs, which were used in order to optimize the SERS performance and enable the ultrasensitive detection of model molecules; this was achieved by tuning the plasmonic couplings and the surface coverage of the NPs. The research was innovative in the determination of the physicochemical properties of the Langmuir monolayers and Langmuir–Blodgett layers formed by the pegylated Au-NRs. Previously unreported dendrimer-like aggregates of the Au-NRs were observed on the solid substrate. Studies have shown that Au-NRs layers increase the intensity of the signals for both substances, which thus provide a high potential for the detection of low concentrations of molecules. Besides the difficult and complex protocols for the design of SERS nanoplatforms, a recently proposed facile protocol was offered for the transfer of Au and Ag NPs of different shapes and sizes, from water into various organic solvents; this was given in the work by Serrano-Montes et al. [29]. The authors of this research optimized the ratio of the commercially available PEG-SH and 1-dodecanethiol to form a stable dispersion of the NPs in non-aqueous solutions. The close packing of the Au-NRs (and other plasmonic NPs) favored plasmon couplings and, thus, provided enhanced plasmonic features. Averaged SERS spectra of 4-mercaptobenzoic acid (4-MBA) for different NP shapes were shown in Figure 2.

### 3.2. Star-Shaped Nanoparticles

Another group of anisotropic NPs that are used as SERS signal enhancers are NSs. They consist of a spherical core attached to branched, acute tips. The presence of numerous acute tips creates many hot spots, and thus the strong field enhancement arises that produces their main advantage [68,69]. Such a structure provides improved optical parameters in comparison to spherical NPs or NRs [70,71]. An interesting aspect of the functionality of Au-NSs is the effect of the branch lengths of the NSs on the enhancement of the SERS signal. According to a study by Mers et al. [72], the short-spiked nanostars (SSNSs) produce greater enhancements of the SERS signals compared to structures with long spikes (LSNSs). This result is surprising because hot spots are located at sharp edges and tips; hence, it was expected that the longer the edges, the greater the signal enhancement. This effect was confirmed by analysis of the SERS spectra of analytes such as glutathione (GSH), which are characterized by weak Raman activity and crystal violet (CV), a Raman-active dye. Glass and indium tin oxide (ITO) were used as the substrates. For glass substrates, the Au-NSs were applied directly to the surface; for ITO substrates, chemical immobilization with the SAMs of 3-mercaptopropyltrimethoxysilane (MPTMS) was necessary. Since the reaction occurs during the immobilization of the surface of the functionalized ITO, MPTMS thiol end groups become available, which are necessary for the binding of the Au-NSs [73]. Therefore, Au-NSs were used as SERS biosensors for the detection of GSH in biological samples. For this purpose, human serum samples were applied onto both SSNS-ITO and LSNS-ITO, and they were analyzed. Studies have shown that the binding of GSH with Au-NSs occurs due to the formation of strong Au-thiolate bonds. In addition, the intensification of the SERS signal occurs due to the chemical interaction of the analyte with the Au-NSs and its surface plasmonic interaction with ITO. In conclusion, the research confirmed the possible use of this type of substrate in biological studies.

**Figure 3:**
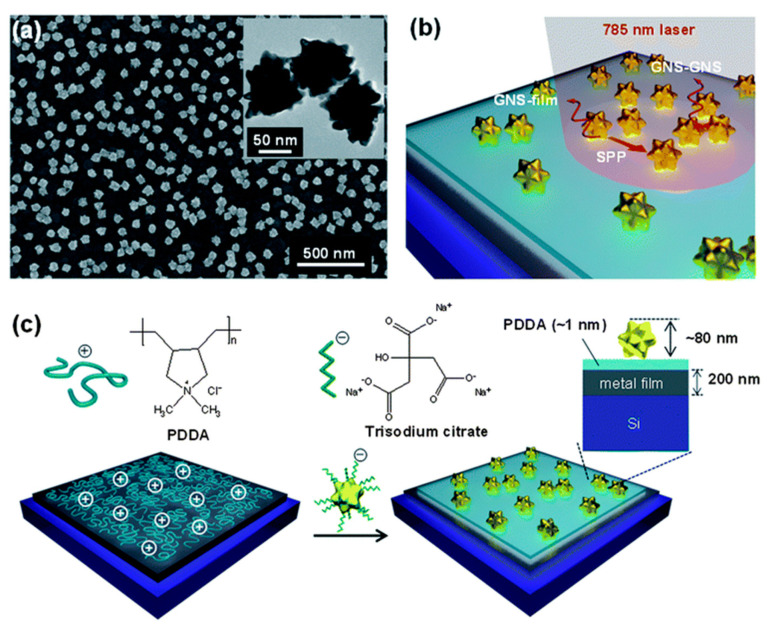
Formation of high-density nanostar (NS) assemblies on metal films. (**a**) An SEM image of high-density NS assemblies on a metal film. The inset shows a TEM image of NSs with sharp tips. (**b**) Schematic representation of field enhancements on the high-density NS assemblies on a metal film showing the NSs-film and NSs-NSs plasmon couplings, in combination with the propagating SPP (surface plasmon polaritons) modes on the metal film. (**c**) Schematic procedure of the electrostatic assembly of high-density NSs on metal films via the electrostatic interaction between negatively charged sodium citrate additives on the NS assemblies and the positively charged PDDA (poly(diallyldimethylammonium chloride) coating on the metal film. Reproduced with permission from [81], Nanoscale; published by The Royal Society of Chemistry, 2014.

A different effect with respect to the influence of branch lengths on SERS efficiency was obtained by Su et al. [74]. Their research showed that the greatest enhancement arises for substrates consisting of NSs with the longest branches. Two different substances were tested as model particles, i.e., Nile blue A and R6G; the substrate used was ITO glass functionalized with 3-amino-propyltriethoxysilane (APTES) on Au-NSs. During the adsorption of Au-NSs on the ITO surface, a probable aggregation of NPs was observed, which resulted from either the high concentration of Au-NSs in the solution or the polarization of gold NSs caused by the positively charged amino group of APTES [75]. Analysis of the SERS spectra also confirmed that the substrates consisting of Au-NS aggregates are effective for the detection of both analytes. The ability to easily change the properties of the NSs (such as the arrangements on its surface) means that NSs are a highly valued material for producing SERS substrates in many different fields. One of their uses is the detection of molecules of both biological and environmental importance. Significantly, the SERS method enables the detection of toxic heavy metals that can be present in the environment. Ma et al. [76] used the self-assembled Au-NS dimers to detect mercury ions (Hg^2+^), one of the most toxic metals. In this work, the Au-NSs were functionalized with thiol-modified DNA and 4-ATP was used as the reporter molecule. The research analysis showed that the Au-NS dimers enhance the intensity of the SERS signal. The reason for the increased intensity of the electromagnetic field may be due to the connection of multiple ends between pairs of molecules, which results in more hot spots compared to individual NPs. Moreover, the nanoplatform used allowed for the detection of Hg^2+^ at the level of 0.8 pg·mL^−1^. Research by Lin et al. [77] also focused on the detection of toxic substances. The authors used Au-NSs, with a size of 40 nm, to quantitatively detect bisphenol A (BPA), which is an endocrine disrupting chemical and a suspected carcinogen [78,79,80]. The innovative approach uses a binary system that involves a SERS lateral flow assay (SERS-LFA). Obtaining innovative SERS sensors of this type can be considered a two-step process. In the first stage, Au-NSs were covered with 4-ATP, which increases the intensity of SERS signals due to absorptions on the gold surface. Then, an anti-BPA antibody was applied to the prepared system, which served as a biorecognition reagent. The reaction mechanism was based on the physisorption of antibodies onto the surface of NPs via electrostatic interactions. Via the application of various concentrations of BPA (from 0.05 to 60 ppb), it was possible to find the detection limit of the substance and, simultaneously, to determine the usefulness of the produced biosensor. The obtained results confirmed the assumed high potential of the produced substrates: a several-fold increase in the intensity peak on the SERS spectra was seen in comparison to spherical NPs. Moreover, the developed SERS-LFA system is characterized by both a wide concentration range and high sensitivity, as evidenced by the detection limit of 0.073 ppb. These approaches to detection, alongside applications for other harmful substances, enable the development of novel, versatile, and highly sensitive sensors that detect trace amounts of materials. Another example of a SERS sensor was fabricated by Lee et al. [81], who aimed to detect trace quantities of dangerous substances. The authors first investigated the plasmonic properties of the Au-NS assemblies that are arranged on the substrates of various metals (e.g., Ag and Au) and dielectrics (e.g., silicon and glass). The reaction between the Au-NSs and the substrate involved an electrostatic interaction between the negatively charged sodium citrate, which was added into the Au-NS solution, and the positively charged polydiallyldimethylammonium chloride layer on the substrates; this is shown in Figure 3. The SERS study enabled determination of the optimal composition of the platform. An analysis of the Raman spectra revealed that the Au-NS assemblies on the Ag films created the highest enhancements. It was found that multiple field enhancements from the interparticle and particle-film plasmonic couplings [58,82] and the lightning rod effects [59] of the sharp tips of NSs are responsible for the enormous Raman enhancements that are seen. Therefore, for example, the SERS sensor presented by these authors can be used to detect trace amounts of nitroaromatic explosives, such as 2,4-dinitrotoluene at the attomolar level.

An important and necessary medical issue is the constant search for new, effective methods of cancer diagnosis and therapy. Research on the use of Au-NSs as contrast agents in the detection of ovarian cancer cells (SKOV3) was performed by D’Hollander et al. [52]. This research focused on determining the properties and potential of SERS-labeled Au-NSs, which have the ability to target cancer cells in vivo. An important element that is responsible for the accuracy and effectiveness of the research was the functionalization of the Au-NSs with a Raman-active label; the molecule used was DTNB, which is characterized by a high reactivity to the gold surface [83,84]. Additionally, in order to increase the stability of the Au-NSs, and to enable further research under physiological conditions, the Au-NSs were functionalized with SAMs. An important parameter for establishing the applicability of Au-NSs in effective SERS imaging is the detection limit of the NSs on a silicon wafer and within agar phantomes. In this case, the physiologically stable SERS-labeled NSs showed a consistent SERS signal for concentrations up to 12 µM. The obtained SERS spectra also confirmed the accumulation of Au-NSs within organs and allowed the detection of concentrations of up to 4.4 pg Au/cell. Such information enabled the authors to proceed to the next stages of the research, which consisted of detecting the SERS signal from the Au-NSs in the liver and tumor of a mouse that contained the SKOV3 cancer cells. To confirm this effect, the accumulation of the Au-NSs in organs was also tested ex vivo. From these results, the Au-NRs were verified as a potential material for cancer diagnosis using the SERS technique. Another possibility when using SERS imaging was presented by Indrasekara et al. [85]. These authors used Au-NSs immobilized on a gold substrate to detect analytes, regardless of their chemical affinity towards gold NPs. SERS substrates were obtained by depositing the layer of Au-NSs onto a silicon substrate, and then immersing the substrate in 6-aminohexanethiolene (AHT). The result of this synthesis stage was the creation of the self-assembled monolayer of AHT, and its task was to immobilize the Au-NSs on the Si wafer; hexane-1-thiol was used as a blocking agent. In order to determine the lowest detection limits, analytes of various concentrations were applied to the prepared substrate. In addition, substances with different chemical structures were used, i.e., 4-MBA, which is a thiolated aromatic molecule, and non-thiolated CV dye. The research proved that the produced nanoplatforms enabled the detection of femtomolar amounts of 4-MBA and picomolar concentrations of CV.

In the case of NSs, as with other shaped NPs, their bimetallic structures can be utilized as SERS sensors. One of the most common schemes of this type is the core-shell system. Here, Ag-coated Au-NSs are characterized by an enhanced SERS efficiency when compared to monometallic systems [86,87]. High-performance SERS substrates that consist of core-shell bimetallic structures were proposed by Tan et al. [88]. Their innovative approach was to assemble the Au@Ag-NSs on the SMCSL superhydrophobic platforms, which was based on an evaporative assembly technique. The close packing of the NPs over the entire region of the superhydrophobic surface resulted in a high density of hot spots, which was verified by the greater enhancements seen in the SERS spectra of the model substances, i.e., Nile blue A, o-phenylenediamine, 6-thioguine, compared to systems consisting of one type of metal (Au-NPs/SMSCL). The obtained results also show that the SERS substrates with such compositions can act as a functional tool for detecting trace amounts of environmental pollutants and drugs. The Au@Ag-NS systems were also investigated by Kaur et al. [89] who demonstrated, using plasmon coupled bimetallic Ag coated Au-NSs dimers containing a controlled nanogap and rectangular DNA origami, the ultrasensitive SERS-based detection of the bacterial biomarker pyocyanin. The functionalization of Au@Ag-NSs with thiolated DNA oligonucleotides was performed by these authors using the method of salt aging. The obtained compound was then immobilized on a rectangular DNA origami, at a predetermined position, with the assistance of extended capturing DNA sequences. This resulted in the formation of DNA origami monomers and dimers; it also enabled the creation of dimer nanoantennas arranged onto two 70 nm Au@Ag-NSs on a rectangular DNA origami with an average interparticle gap size of 10 nm. Such SERS sensors have the potential to detect biomarkers at the single-molecule level, as evidenced by the detection of pyocyanin at the concentration level of 335 pM. Another similar type of system also has substrates that consist of 2 derivatives of NPs. One example of such is SERS sensors that consist, simultaneously, of two derivatives of Au-NSs, as presented by Pei et al. [90]. These authors used the densely packed self-assembling substrates of the Au-NSs and the labeled immune NS aggregates. The formation of these nanoplatforms was based on the self-assembly between polyelectrolytes and the Au-NSs, which occurs due to their electrostatic interactions. To produce this system, glass slides were immersed several times within the Au-NSs and polystyrene sulfonate solution. This enabled the development of a highly sensitive sandwich immunoassay, the purpose of which is the identification and quantification of specific antigen-antibody bonds via the SERS technique. The applied substrates are characterized by improved detectability compared to substrates that consist of spherical NPs.

### 3.3. Prism-Shaped Nanoparticles

Noble metal NPRs are another group of NPs that are widely applied in SERS schemes. Identical to the previous cases, the plasmonic properties of the NPRs are influenced by their morphological parameters, such as their edge length or tip rounding. The mutual arrangements of the NPR particles influence the presence of the plasmonic hot spots, which are found in tip-to-tip and edge-to-edge patterns. In the case of NPR arrays, the greatest electromagnetic field enhancements are usually seen between the tips of adjacent particles [91,92]. Assembled structures of NPs with a large number of hot spots can be obtained using the Langmuir–Blodgett or Langmuir–Schaefer techniques. This involves the creation of packed monolayers of NPs at the water/air interface, which is then transferred to a solid substrate. Using this method, assembled structures with a given packing are obtained [93,94]. The Langmuir–Schaefer technique is used to fabricate over a large area near to the close-packed Au-NPR monolayers, which were then deposited onto dielectric and mirror substrates as described by Lee et al. [95]. The CTAB-coated Au-NPRs used in this research were functionalized by the use of PVP, which enhanced the ability of the Au-NPRs to be dispersed within organic media. The self-assembled platforms formed on the solid substrate (Si purified with oxygen plasma) were characterized by the close-packing of the Au-NPR particles, which simultaneously created ordered structures. A significant number of hot spots were recorded for the tip-to-tip and edge-to-edge inter-particle regions, which resulted in an enhancement of the local electromagnetic field, as seen in the SERS spectra of 2-naphthalenothiol. An additional advantage of these nanoplatforms was the lack of any significant rounding at the particle edges throughout the entire assembly, which also contributed to an enhanced SERS signal. An innovative approach that was applied in this research was the transfer of the two-dimensional arrays of ultra-thin Au-NPRs to a metallic film, which served as a mirror to create a conjugated image of the charge distributions of the NPR monolayer on it. Moreover, in this case, there was significant enhancement of the SERS, which may be caused by the interactions between the surface plasmons of the NPs and the propagating plasmons of the thin-metal film underneath the NPs. The procedure for producing the nanoplatforms at the air/liquid interface, using Au-NPRs functionalized with PVP, was also proposed by Scarabelli et al. [96]. The Au-NPR monolayers can be obtained after the dispersal within an ethanol–hexane solution, which was then transferred onto a glass substrate using the Langmuir–Schaefer technique; this enabled the self-assembly of the required particles.

To characterize the properties of the material, SERS studies were performed on the analytes, i.e., BT and CV at different concentrations. Due to the different mechanisms for the interactions of these substances with the SERS sensor, a variety of results were observed. In the case of the BT, it was found that the detection limit for a solid substrate is an order of magnitude higher than for the solution. For the CV, in contrast, the type of substrate had no effect on the detectability. Walker et al. used various types of substrates in their research [97]. These authors produced an assembly of Au-NPRs, functionalized with SAMs of N,N,N-trimethyl(11-mercaptoundecyl)ammonium chloride (TMA), which were ordered into monolayer and multilayer structures. The assembly of NPRs was performed on solid substrates, such as monocrystalline silicon wafers, oxidized silicon wafers (Si/SiO_2_), gold-plated silicon wafers functionalized with TMA SAMs, and gold-coated silicon wafers functionalized with mercaptoundeconic acid SAMs. The SERS studies that were conducted showed that the proposed structures were able to increase the intensity of the methylene blue signals by an order of magnitude. This confirmed the possibility of using the fabricated structures as specific SERS sensors, due to the precise arrangements that can be achieved by adjusting the repulsive interactions between the particles.

**Figure 4:**
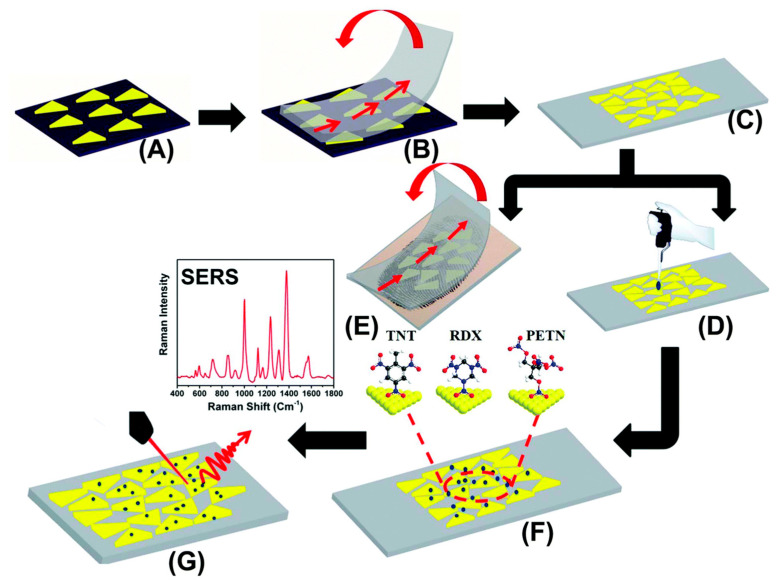
Design of Au-NPR (gold nanoprism)-based SERS nanosensor for detection of trace explosives: (**A**) a self-assembled layer of Au-NPRs on an APTES (3-aminopropyl)-triethoxysilane) functionalized glass coverslip was prepared. (**B**) Au-NPRs were transferred to a flexible adhesive substrate by the stamping technique, which produced a SERS nanosensor (**C**). Explosive molecules were either drop-casted from a solution (**D**) or transferred from a thumb impression (**E**) directly onto the SERS nanosensor (**F**). (**G**) SERS spectra were collected using benchtop Raman spectrometer at a 785 nm diode laser excitation. The fabrication approach of SERS nanosensor is a schematic representation; none of the figures present an exact number and/or density of Au-NPRs in each step. The image is not to scale. Reproduced with permission from [98], Analyst; published by The Royal Society of Chemistry, 2018.

An interesting method for creating SERS sensors composed of Au-NPRs, which is used for detecting explosives, was presented by Liyanage et al. [98], and it is presented in Figure 4. The nanoplatforms that were produced consisted of self-assembled Au-NPRs upon a flexible adhesive surface. The procedure for forming the SERS nanosensors involved the depositing of Au-NPRs onto a functionalized APTES glass substrate, which formed a self-assembled layer that was then transferred onto adhesive tape using a stamping technique. The use of this nanosensor for SERS measurements verified its usefulness in the detection of trace amounts of explosive substances. A significant advantage of the presented nanoplatforms was its high sensitivity, which ensured the detection of substances at a level as low as 56 ppq. The potential use of the Au-NPRs as nanoplatforms to be used in Raman spectroscopy was also presented by Liebig et al. [99]. These authors offered a method for producing close-packed, self-assembled Au-NPR monolayers on solid substrates, such as silicon wafers or quartz glass, that rely upon the transfer of particles at the air/liquid interface following the addition of a toluene-ethanol mixture. The dimerization of the 4-NTP into 4,4′-dimercaptoazobenzene was used as a model reaction that confirmed the photocatalytic activity of the tested material. In order to investigate the dynamics of the dimerization reaction, the 4-NTP monolayer was assembled onto the Au-NPRs, and then a SERS analysis (as a function of time) was performed. This enabled the confirmation that the Au-NPRs can act as reaction catalysts. The mechanism for the latter is based upon the production, by the Au-NPRs, of hot spots that initiate the dimerization process via the molecular orbitals of the adsorbed 4-NTP. In addition, this SERS technique proved to be an effective tool for the monitoring of reaction dynamics in real time.

As with other shaped NPs, bimetallic systems are also possible for NPRs. The plasmonic properties of such core-shell systems (Au@Ag-NPRs) were investigated by Liebig et al. [100]. In this work, Ag-NPs were synthesized on the surface of Au-NPRs and stabilized with sodium dioctylsulfosuccinate (AOT) and benzylhexadecyl-dimethylammonium chloride (BDAC). The synthesis of Ag-NPs was made possible due to the presence of the AOT/BDAC layers that hindered the transport of the Ag^+^ ions. The material prepared by this procedure was then self-assembled on the glass substrate, by adding the toluene-ethanol mixture that wetted the substrate and enabled the creation of the shimmering layer at the interface. The potential application of the prepared nanoplatform using SERS schemes was investigated with the dye R6G. Moreover, the self-assembled analyte molecules on the nanostructure monolayers were observed during the SERS analysis. It was determined that the signal enhancement is heightened for greater quantities of Ag-NPs in the Au@Ag-NPRs. This can be explained because Ag-NPs form nanogaps, which produce the additional hot spots that are responsible for the enhanced signal.

### 3.4. Cube-Shaped Nanoparticles

NCs are also an important group of NPs that are widely used in SERS schemes. For NCs, the plasmonic properties depend on the gap between adjacent nanostructures. It has been determined that ordered 2D structures on solid substrates can contain high SERS enhancements [101,102]. These types of arrays can again be obtained using the Langmuir–Blodgett technique. Ag-NC monolayers are excellent candidates for the SERS quantitative analysis of molecules. Substrates relating to high-performance SERS have been prepared, via the water/oil interfacial transfer method, by use of Ag-NCs, polyelectrolyte, and gold film. Ag-NCs have a large number of hot spots, but it is the spaces between the NPs that lead to SERS signal enhancement. This indicates that the self-assembled Ag-NCs are effective as SERS substrates used in the analysis of complex molecular systems. The dyes R6G and CV generate characteristic SERS signals with varying intensities, which enables the simultaneous detection of double molecules [103]. Ag-NCs deposited on the surface of a quartz substrate can operate using the technique demonstrated by Mahmoud et al. [104]. In this work, the Ag-NCs dispersed within chloroform were applied to an aqueous subphase, and then the monolayer was compressed under different surface pressures. The surface film that was prepared in this method was transferred to a quartz substrate, which enabled it to be tested as a SERS sensor. PVP was used as the SERS probe and the capping agent during the synthesis of the Ag-NCs. This research showed that the signal enhancement that appears in the SERS spectra is dependent on the amount of surface pressure applied during the deposition; the SERS intensity increases under a greater pressure, which arises because of the growth in the aggregation of the Ag-NCs at higher pressures. Therefore, it was determined that the aggregation of the particles in the monolayer is a beneficial phenomenon for increasing SERS, as visualized by the broad LSPR band that represents a strongly enhanced signal intensity.

The Ag-NCs have also been used in the fabrication of hybrid SERS substrates, as presented by Banchelli et al. [105]. Ag-NC nanosystems that are synthesized in the presence of PVP were covered with a graphene oxide layer (GO/Ag-NCs) via its spontaneous adsorption. Monolayers of the Ag-NCs were assembled on the surface using two different procedures—Langmuir–Blodgett transfer to a glass or silicon substrate at different surface pressures and then sequential self-assembly by physisorption onto the surface; this enables the determination of the SERS activity, which depends on the research approach. SERS experiments were performed using the model probe adenine because of its well-known SERS response and the potential to establish interactions between the noble metal and graphene surfaces [106]. Based on this method, it was found that the GO/Ag-NC nanoarrays have a greater potential for application as SERS sensors than systems that consist of Ag-NCs. Moreover, hybrids obtained from the Langmuir–Blodgett technique were characterized by a higher Raman enhancement, which means it is more useful for SERS research.

NP clusters that are composed of self-assembled Ag-NCs can be used as substrates in SERS. In such systems, the SERS signal can be enhanced by considering the structure of the cluster and the measurement conditions. Ag-NCs can be grouped into small clusters in circular positions on the substrate, which enables a study of the structure and the plasmonic properties of a large number of NP clusters. The differences in the cluster-to-cluster SERS factors were analyzed and correlated with both the cluster size and the laser frequency. It was determined that an increase in the mean gain values can occur by increasing the number of Ag-NCs in the cluster to 4 Ag-NCs per cluster. Moreover, the most effective SERS substrates have been shown to be the linear aggregates, in which Ag-NCs are attached along the edges. A direct analysis of the SERS enhancement distribution provides a lot of information on the preparation of the substrates that are composed of Ag-NC clusters. Additionally, the distribution of values of the enhancement from clusters of the same size becomes narrower with an increase in the cluster size. However, to date, no correlation has been established between the maximum enhancement value and the size of the cluster [107].

Nanoplatforms based on at least two noble metals are an important material in SERS research. The core-shell bimetallic system was used in the research by Lin et al. [108]. These authors prepared the platforms using Au@Ag-NC self-assembled monolayers with embedded 4-MBA, which acted as an internal reference between the gold and silver surfaces. The prepared monolayer films were then transferred to either silicon wafers or a glass substrate. A significant element of this research was the precise determination of the edge lengths of the Au@Ag-NCs, which enabled the optimization of the SERS performance. To test the properties of the produced substrates, the SERS analyses were performed using CV and aspartame as the analytes. The signal enhancement that appears in the relevant spectra confirmed the usefulness of the sensors as SERS enhancers. An innovative element of the SERS substrates is the use of filter paper, as proposed by Lin et al. [109]. Using a liquid/liquid interface self-assembly technique, these authors created a nanoplatform via the assembly of Au@Ag-NCs onto a patterned filter paper, with the assistance of a mask paper. The prepared material was glued to a glass slide by the use of poly(methyl methacrylate) double-sided tape, and then its practicality was tested for SERS applications. Another innovative concept is the possibility of the simultaneous extraction and detection of the analytes in complex samples, due to the presence of the sample injection zone and the detection area on the SERS platform. Analysis of the SERS measurements performed for 4-MBA showed that the employed substrate had excellent reproducibility. This enabled an accurate quantitative analysis of the SERS signal from thiram, which is a fungicide that can be present in soil. The obtained results, using the SERS technique, showed the excellent capabilities of the tested system for the detection of pesticide residues.

Si et al. [110] presented self-organizing Au@Ag-NCs as the SERS substrates for quantitative drug identification on surfaces. Au@Ag-NC plasma nanosheets are mechanically soft and optically translucent, which allows their conformal attachment to solid surfaces, such as banknotes, for drug identification. This system was able to detect benzocaine at ppb concentrations, and several other drugs can also be identified, even in mixtures. Additionally, even at very low concentrations, such system can be used to detect illicit pharmaceuticals. The advantage of this detection system based on SERS is the low cost, high sensitivity, and a direct qualitative and quantitative analysis without the need for subsequent sample processing steps. Ultra-soft Au@Ag-NC plasma nanosheets are a new class of SERS platforms that is used for identifying drugs on solid surfaces [110]. Another effective solution for the production of sensitive SERS substrates is the use of multilayer structures that consist of several metals. A possible nanoplatform of this type is a Ag-NCs/polyelectrolyte/Au (Ag/PE/Au) sandwich-structured film, which is presented by Wang et al. [111]. The fabrication of the Ag/PE/Au film involved layer-by-layer deposition. In the initial step, a Au film with a Cr adhesive layer was applied to the Si substrate, which was then immersed into a cationic or anionic polyelectrolyte solution. The material was then coated with Ag-NCs and used as the SERS substrate. In this research, the dyes R6G and CV (at different concentrations) were used as the probe molecules. For both analytes, it was determined that the presented system had high sensitivity compared to systems that consist of Ag-NCs. It was also found that this sensitivity depends on the thickness of the PE layer; the maximum signal was recorded for cases with three layers of PE. Additionally, the Raman spectra showed a signal enhancement, which proved that the Ag/PE/Au film had excellent SERS enhancing properties. The reason for this can be explained by the coupling of the localized surface plasmons, and the surface plasmon polaritons resonances, between adjacent Ag-NCs and between the Ag-NCs and the Au film. A similar effect could be observed in plasmonic gratings for which the effect of incident light plays a crucial role [112,113].

## 4. Assemblies of Noble Spherical Nanoparticles

Noble spherical NPs are the most widely used NPs in SERS applications. Single NPs exhibit moderate Raman signal enhancements, while aggregated NPs have very strong enhancements due to the three-dimensional distribution of the plasmonic hot spots. The performance of the NP assemblies depends strongly on their surface coverages. NPs begin with relatively poor SERS efficiencies in dilute layers, but they undergo a large boost in the accumulation of hot spots when surface coverage increases, which ensures a higher SERS efficiency [56]. Metal NP assemblies can be varied by simply changing the chemical design parameters in a manner that optimizes the spacing and the cluster arrangements of the spherical NPs. In the work of Adams et al. [114], Au-NPs were attached, via a crosslinking reaction, to chemically functionalized poly(methyl methacrylate) domains on polystyrene-block-poly(methyl methacrylate) templates. The Au-NPs could be arranged into clusters by changing the type of crosslinking agent that is used or by varying the concentration of the functional groups. Due to the chemical immobilization of the Au-NPs on the polymer template and the small interparticle spacings, a high surface coverage over the cluster assemblies was obtained. For these types of Au-NP arrangements, a uniform, highly enhanced Raman signal was obtained with an increase on the order of 109. Gehan et al. [115] presented the covalently bound functionalization of Au surfaces by use of functional aryl groups, which are derived from the electroreduction of diazonium salts and a subsequent derivatization to obtain azide-terminated layers. At the surface of the gold electrodes, the Au-NPs were easily self-assembled, and thus, the Raman enhancement was strong for these Au-NP assemblies coated on gold electrodes; this originated from the strong electromagnetic coupling between the NPs and the substrate. Two of the most versatile methods, which can be used to control the arrangements of the NPs in two dimensional layers, are the Langmuir and Langmuir–Blodgett techniques. Ishida et al. [116] provided a simple approach for surface modifications with hydrophobic thiol, or its dye-derivative of hydrophilic citrate-capped Au-NPs with a 50 nm size. The authors investigated several organic solvents, in which the citrate-capped Au-NP, thiols, and porphyrin used for the modification were all soluble, in order to select the appropriate solvent to create a one-step surface-modification procedure. Two-dimensional monoparticle films with surface-modified Au-NPs were prepared using the Langmuir–Blodgett method. The SERS nanoplatform that was prepared by this method greatly improved the Raman signal from cooper phthalocyanine.

**Figure 5:**
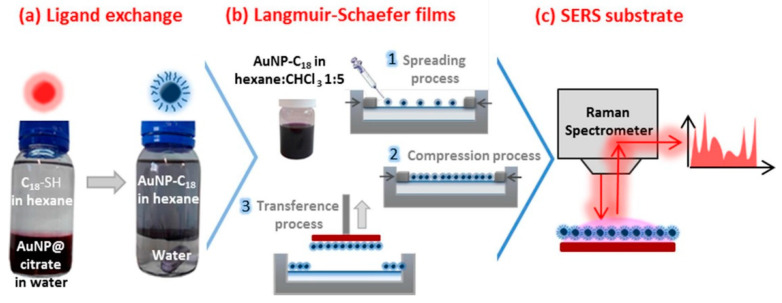
Scheme showing the protocol followed in the work by Lafuente et al.: (**a**) preparation of the gold nanoparticles dispersion, (**b**) fabrication of Langmuir films and Langmuir–Schaefer modified substrates incorporating octadecylthiolate capped gold nanoparticles, (**c**) application of these substrates for SERS detection. Reproduced with permission from [53], Applied Surface Science; published by Elsevier, 2020.

Further work on the functionalization of spherical NPs was completed by Serrano-Montes et al. [29]. These authors optimized the transfer of the noble metal NPs from water into organic solvents; this was achieved by the careful selection of a combination of the PEG-SH and a hydrophobic capping agent, such as dodecanethiol. The NPs prepared using this procedure were characterized by the long-term stability of the organic dispersions; moreover, the procedure led to the self-assembly of the extended NP arrays at the air/liquid interface, which is transferred onto solid substrates and used as a SERS substrate. These types of substrates can be highly efficient due to the dense close packing of the obtained NP assembly, which results in extensive plasmonic couplings. The approach recently proposed by Serrano-Montes et al. [29] was used in the work by Tahghighi et al. [117]. This produced a minimization of the unwanted aggregation of the Au-NPs by the combination with commercially available organothiols, which allowed the dispersion of the Au-NPs in a volatile solvent and enabled a spreading of the spherical NPs at the water/air interface. Moreover, the SERS enhancement for the reference analyte can be tuned by controlling the lateral surface pressure of the Langmuir monolayer; thus, a tailoring of the packing density of the Au-NPs at the water/air interface is achieved. Beside the Langmuir–Blodgett technique, homogenous SERS substrates could be prepared using the Langmuir–Schaefer technique. The latter was presented in recent work by Lafuente et al. [53]. Homogeneous tightly packed films with octadecylthiolate capped Au-NPs (with 27 nm diameter), which were produced in a reproducible and simple manner, were obtained. The fabricated Au-based SERS substrates that resulted were able to detect R6G in 10–11 M aqueous solution and had a remarkable spatial uniformity for the SERS response over the scanning area. The Langmuir–Schaefer deposition enabled a high reproducibility from batch to batch, with a standard deviation below 10% for the SERS signal. The Au-NPs larger than 20 nm diameter may greatly enhance the Raman intensity, if they can be assembled into ordered monolayers. The scheme for the protocol, which is followed by Lafuente et al. [53], is shown in Figure 5.

Recently, a three-phase self-assembly method was presented by Yang et al. [118], which resulted in the successful ordering of the Au-NP monolayers for particle diameters that range from 13 to 90 nm. This technique included a water/oil/air phase and used a commercially available alkylamine ligand to stabilize the self-assembled Au-NPs at fixed separations. The assembled layers had long-range orderings and contained a local hexagonal close-packed structure. For the optimized arrays, and with R6G as the model analyte, high values for SERS enhancements were achieved, which were as high as 107 times larger. Yang et al. [55] used the water-organic solvent self-assembly strategy to produce 2D Au-NPs monolayer films. Moreover, the Au-NP assembly can be tuned into the single-figure nm regime by use of alkylamine rather than alkylthiol ligands of different lengths. These authors also stated the positive effects caused by the alkylamine ligands during the 2D self-assembly of the Au-NPs. Nanoplatforms that consist of Ag-NPs are also an effective SERS sensor, as demonstrated by Fan et al. [119]. They proposed a self-assembled substrate composed of Ag-NPs and a MPTMS sol-gel. The procedure for the formation of the SERS platform involved a modification of the glass substrate using MPTMS and then a depositing of the Ag-NPs. An important element of this process was the use of a specific number of NPs on the surface of the substrate, which was obtained by dipping the plate several times into the Ag-NP solutions and the MPTMS sol-gel. The mechanism for the formation of subsequent layers was based upon the interaction of the Ag-NP with the pendant thiol groups of the MPTMS. The suitability of the prepared material was examined by performing a SERS analysis using Nile blue A and oxazine 720. As a result, it was found that the proposed nanoplatforms contained good plasmonic properties, which was justified by the enhanced SERS signal. After the depositing of seven Ag-NPs, a system with the highest enhancement was achieved.

An outstanding nanotechnological tool for SERS nanoplatform design is based upon DNA molecules [120]. DNA has a great ability to assist the assembly of spherical NPs into well-defined materials. A general strategy is centered upon DNA origami, in a process that involves the folding of DNA to create 2D and 3D objects at the nanoscale. In the simplest stable scenario, a biocompatible SERS-active system could be obtained by the covalently bonded functionalization of Au-NPs with specific DNA strands, as described by Caprara et al. [121]. The mesoscopic aggregation of Au-NPs functionalized with 12-base single-stranded DNA sequences were induced by the addition of a complementary 24-base bridge sequence. The aggregation process of the Au-NPs was induced by the bridged DNA molecule, which resulted in a more pronounced SERS spectra reproducibility. In contrast, a lack of signal reproducibility was observed for single DNA functionalized Au-NPs, in which the aggregation process is both spontaneous and uncontrolled, and thus, many effective hot spots could not be induced. The SERS enhancement factor can be increased by selecting shorter DNA sequences for such Au-NPs systems. A more complex scheme was recently provided by Zhou et al. [122], which involved a study on a DNA origami template. They expanded the structural complexity of the SERS-active metamolecules by increasing the number of Ag@Au core-shell NPs from 4 to a maximum >30. The interparticle gaps in the hexagonal arrangement of the Ag@Au-NPs were strongly dependent on the silver growth time. The gaps <5 nm produced the strong plasmonic hot spots that are useful for Raman enhancements; this was achieved for a silver growth time of 10 min.

Huge Raman enhancement effects arising from the hexagonal monomer metamolecules were derived from an adaptation of the spatial arrangements of the NPs. Aggregation of the Au-NPs during the aging process may result in an unwanted reduction in the active surface area. Such aggregation, which deteriorates the particles used as SERS substrates, can be avoided by the fixation of the Au-NPs into one-dimensional (1D), 2D, or 3D spaces. Chen et al. [123] proposed a surfactant-free synthesis of 3D Ag microspheres in an aqueous phase. The 3D microspheres act as supports for fixing the Au-NPs into 3D space via the interactions between the carboxyl groups of the Au-NPs and the Ag atoms of the microspheres. The SERS activity of the Au-NP assemblies at the microspheres is dependent on the ratio of Au-NPs to the microspheres; this relates to the decreased nanogaps seen with an increased Au-NP content on the surface of the microspheres. Another example of 3D assemblies for SERS applications was described by Xia et al. [124]. In this research, Au-NPs were assembled on Ag spheres by a replacement reaction process between chloroauric acid and the Ag spheres. The Ag particles decorated by the Au-NPs were always multilayered, which offered a larger specific surface area and provided more hot spots within the input laser excitation area. Besides the described systems, the NPs could be assembled with the use of highly anisotropic gold nanowires. In this type of scenario, one kind of NP induced the spatial distribution of others. Sánchez-Iglesias et al. [125] showed binary self-assembly of gold nanowires and NPs. Long-length gold nanowires can induce an assembly of spherical Au-NPs into ordered arrays, when oleylamine is present on their surfaces and in the solution used. Moreover, nanowires can be employed to tune the distances between the NPs, thereby altering the overall optical response of the film.

## 5. Conclusions and Perspectives

Metallic nanoparticles (NPs) that are synthesized using bottom-up approaches have a wide variety of potential applications. NPs exhibit a range of unique physiochemical characteristics, and they can contain excellent optical properties. In particular, NPs can be used as enhancers in surface-enhanced Raman spectroscopy (SERS). Such features suggest that NPs are promising nanomaterials for use in biomedical applications. In Table 1, we summarized the basic functional properties of NPs and their arranged systems; this is based on the comprehensive literature analysis that is described in this review. The optical properties of the NPs, such as the localized surface resonance plasmon band and their ability for generating plasmonic hot spots, can be tuned by the careful selection of the shape of the NPs and by controlling the quantity of chemical reagents used. However, efficient NPs are typically prepared as hydrophilic NPs that are unsuitable for SERS and, thus, need to be further functionalized using a variety of approaches; different methods have also been used to produce efficient SERS nanoplatforms. Such solutions are mostly based on the careful functionalization of the surface of the NP and the preparation of SERS substrates. The functional SERS nanoplatform, which are obtained via these procedures, are mainly tested using model SERS molecules such as dyes and small molecules containing –SH group. In particular cases, the described nanoplatforms can be expected to be efficiently utilized and commercialized. However, a strong need remains for the exploration of new directions in the research on arranged NPs, which will be pivotal for the future development of highly effective SERS nanoplatforms. Significantly, the use of arranged NP systems based on SERS in biological applications still faces a variety of limitations, which includes a need for a coupling of the analytes to the plasmonic surface, ideally within the plasmonic hot spots. Furthermore, progress towards their widespread adoption in clinical diagnostics is still very limited, due to the use of highly diluted samples in complex matrices and the requirement of advanced instrumentation. Nevertheless, further research on both the functionalization of NPs and the practical biological applications of the SERS nanoplatforms can resolve existing problems. The ultimate aim is the production of direct and indirect breakthroughs, so that SERS nanoplatforms can be applied to everyday life.

## Figures and Tables

**Table 1:** Basic functional properties of the noble metal nanoparticles and their arranged systems.

Property → NPs Shape ↓	Noble Metal NPs Size * [nm]	NPs Dispersion Medium	Type of Ligand at NPs Surface	Substrate Preparation	Type of Arrangement Method Used	SERS Tested Molecule	Ref.
Spherical NPs	Au 20	H_2_O	EDC/S-NHS	Diblock copolymer	Domain ML SA	BT	[114]
	Au nd	H_2_O	Tri-SC	NH_2_ coated Au	ML SA	MB	[115]
	Au 17, 33, 48, 75	CHCl_3_	ODT	SCP	ML LB	CuPc	[116]
	Au 100 Ag 50	EtOH + hexane	ODT + PEG-SH	SCP	ML SA	4-MBA	[29]
	Au 10	CHCl_3_	ODT + PEG-SH	Silinization	ML LB	4-MBA	[117]
	Au 27	CHCl_3_ + hexane	Octadecylthiolate	SCP	ML LS	R6G	[53]
	Au 13, 40, 60, 90	H_2_O	SC	SCP	ML 3 phase assembly	R6G	[118]
NRs	Au 53.2 × 23.6	MeOH + CHCl_3_	PEG-SH	SCP	Dendritic LB	4-MBA, R6G	[67]
	Au 61.0 × 15.0	EtOH + hexane	ODT + PEG-SH	SCP	ML LS	4-MBA	[29]
	Au@Ag 71 × 34	H_2_O	Gemini surfactant	ITO covered glass	3D crystals SA	BT	[62]
	Au 75 × 25	H_2_O	CTAB	Plasma cleaning	3D supercrystals SA	Prion protein	[64]
	Au 53 × 22	EtOH	3-MBA + ODT	SAM covered Au	nd SA	Glucose	[66]
	Au 55.0 × 16.5	EtOH + H_2_O	MUHEG	SCP	Hierarchical SA	Py	[126]
	Au 44.8 × 11.2	H_2_O	CTAB	Photonic crystal on Si	3D on silica NPs SA	R6G	[44]
NSs	Au 60 × nd × nd, 65 × 75–80 × nd	H_2_O	Chitosan	SCP	ML SA	GSH, CV	[72]
	Au 21 × 20 × 12, 22 × 13 × 13, 22 × 7 × 12	H_2_O	HEPES	APTES covered ITO glass	Aggregates surface-assembly	NBA, R6G	[74]
	Au 50 × nd × nd, 60 × nd × nd	nd	Thiol-modified DNA	nd	Dimers SA	4-ATP	[76]
	Au 40 × nd × nd	H_2_O	SC	nd	nd	BPA	[77]
	Au 57 × 11 × 18	NH_2_OH + HEPES	SC	PDDA coated substrates	ML SA	BT, DNT	[81]
	Au 66.3 × nd × nd	H_2_O	SC	nd	ML SA	DTNB	[52]
	Au 36 × 3–5 × nd	H_2_O	Tri-SC	SCP, AHT coated substrate	Domain ML SA	4-MBA, CV, 4-ATP, PODT, NP	[85]
	Au@Ag nd × nd × nd	H_2_O	SC, AA	nd	Dimers evaporative assembly	NBA, OPD, 6-TG	[88]
	Au@Ag nd × nd × nd 70 nm (tip to tip distance)	HEPES	Thiolated oligonucleotides	Plasma cleaning	Dimers SA	Py	[89]
	Au 69.6 × 13 × nd	H_2_O	CTAB	SCP, PEI and PSS solution	multilayers SA	4-MBA	[90]
NPRs	Au 134 × 10	EtOH + hexane	PVP	nd	ML LS	NAP	[95]
	Au 71 × nd	EtOH + hexane	PVP	SCP	ML SA	BT	[96]
	Au 158.0 × 8.7	EtOH	AC	nd	ML SA	MB	[97]
	Au 42 × nd	Acetonitrile	PMHS + TOA	APTES covered glass	ML SA	TNT	[98]
NCs	Ag 70	EtOH + H_2_O	PVP	nd	ML LBL	R6G, CV	[103]
	Ag 50	CHCl_3_	PVP	SCP	ML LB	PVP	[104]
	Ag 45	CHCl_3_	PVP	Plasma cleaning	ML LB, SA	Adenine	[105]
	Ag 85, 110	H_2_O	PVP	nd	Clusters Vertical and electrophoretic deposition	4-ATP	[107]
	Au@Ag 46, 56, 68, 82	H_2_O	CTAC	nd	ML SA	CV, APM	[108]
	Au@Ag 53	EtOH	PVP	nd	ML SA	4-MBA, Thiram	[109]
	Au@Ag 35	PS-THF + CHCl_3_	PS	nd	Plasmene nanosheets SA	Benzocaine, Ibuprofen, AC, aspirin	[110]
	Ag 60, 90, 115	H_2_O	PVP	SCP	Multilayers LBL	R6G, CV	[111]

*—the mean size of the NPs was indicated, for NSs: core diameter × branch length × branch diameter, for NPRs: side length × thickness. Abbreviations: 3-MBA–3-mercaptophenylboronic acid, 4-ATP—4-aminothiophenol, 6-TG—6-thioguine, AA—ascorbic acid, AC—acetaminophen, AC—N,N,N-trimethyl(11-mercaptoundecyl)ammonium chloride, AHT—6-aminohexanethiolene, APM—aspartame, APTES—(3-aminopropyl)-triethoxysilane, BPA—bisphenol A, BT—benzenethiol, CTAB—hexadecyltrimethylammonium bromide, CTAC—Cetyltrimethylammonium chloride, CuPc—cooper phthalocyanine, DNT—2,4-dinitrotoluene, DTNB—5,5-dithio-bis-(2-nitrobenzoic acid), EDC—hydrochloride, EtOH—ethanol, GSH—glutathione, HEPES—N-(2-Hydroxyethyl)piperazine-N’(-2-ethanesulfonic acid), HMX—octahydro-1,3,5,7-tetranitro-1,3,5,7-tetrazocine, ITO—indium tin oxide, LB—Langmuir-Blodgett, LBL—layer-by-layer, LS—Langmuir-Schaeffer, MB—methylen blue, MeOH—methanol, ML—monolayer, MUHEG—(11-mercaptoundecyl)hexa(ethyleneglycol, NAP—2-napthalenethiol, NBA—Nile blue A, NCs—nanocubes, nd—not determined, NP—naphthalene-2,6-dithiol, NPRs—nanoprisms, NPs—noble metal nanoparticles, NRs—nanorods, NSs—nanostars, ODT—octadecanethiol, OPD—o-phenylenediamine, PEI—polyethyleneimine, PMHS—poly(methylhydrosiloxane), PODT—5-phenyl-1,3,4-oxadiazole-2-thiol, PS—thiolated-polystyrene, PSS—polystyrene sulfonates, PVP—poly(vinyl pyrrolidone), Py—pyocyanin, RDX—hexahydro-1,3,5-trinitro-1,3,5-triazine, Ref.—reference, SA—self-assembling, SAM—self-assembly monolayer, SC—sodium citrate, SCP—standard cleaning procedure (different methods used for removing organic compounds, such as RCA1 and piranha etch), S-NHS—N-hydroxysulfosuccinimide, THF—Tetrahydrofuran, TNT—2,4,6 trinitrotoluene, TOA—trioctylamine.

## Data Availability

Not applicable.
